# Cigarette smoke extract induces airway epithelial cell death via repressing PRMT6/AKT signaling

**DOI:** 10.18632/aging.202210

**Published:** 2020-12-01

**Authors:** Tiao Li, Kristen V. Fanning, Toru Nyunoya, Yan Chen, Chunbin Zou

**Affiliations:** 1 Department of Pulmonary and Critical Care Medicine, The Second Xiangya Hospital, Central South University, Changsha 410011, Hunan, China; 2 Division of Pulmonary, Allergy, and Critical Care Medicine, Department of Medicine, University of Pittsburgh School of Medicine, Pittsburgh, PA 15213, USA

**Keywords:** cigarette smoke, cell death, lung epithelia, PRMT6, PI3K/AKT

## Abstract

Chronic obstructive pulmonary disease (COPD) is a severe public health threat world-wide. Cigarette smoke (CS)-induced airway epithelial cell death is a major pathway of pathogenesis in emphysema, a subtype of COPD. Protein arginine methyltransferase 6 (PRMT6) is a type I PRMT that catalyzes mono- and di-methylation on arginine residues within histone and non-histone proteins to modulate a variety of life processes, such as apoptosis. However, its role in CS-induced lung epithelial death has not been fully elucidated. Here we report that PRMT6 was decreased in mouse lung tissues from a cigarette smoke extract (CSE)-mediated experimental emphysematous model and in CSE treated or cigarette smoke exposed lung epithelial cells. Depletion of PRMT6 increased the protein levels of phosphatase PTEN and PI3K regulatory subunit p85 but decreased a downstream kinase PDK1, resulting in AKT dephosphorylation and thereafter, lung epithelial cell death. Knockout of PRMT6 inhibited epithelial survival and promoted CSE-mediated epithelial cell death, while ectopic expression of PRMT6 protein partially reversed epithelial cell death via PI3K/AKT-mediated cell survival signaling in CSE cellular models. These findings demonstrate that PRMT6 plays a crucial role in CS-induced bronchial epithelial cell death that may be a potential therapeutic target against the airway cell death in CS-induced COPD.

## INTRODUCTION

Chronic obstructive pulmonary disease (COPD) is the fourth leading cause of death world-wide, which is defined as a common disorder of progressive airflow limitation with diverse respiratory symptoms [1]. The major etiological risk factors triggering COPD pathogenesis are long-term exposure of the cigarette smoke (CS) and biomass fuel smoke to the airway and/or alveolus [1]. Cigarette smoke contains a concoction of toxic chemicals with a median aerodynamic diameter of 0.45 μm [2]. The smoking particles settle on the surface of the lung epithelial cells in bronchioles and respiratory airways, causing persistent oxidative stress and inflammatory responses, exacerbating the destruction of lung parenchyma and airway remodeling, accelerating irreversible lung function decline, and leading to airflow limitation and chronic respiratory symptoms associated with COPD [1, 3]. Importantly, CS-induced inflammation, featured with substantial lung epithelial cell death, initiates and participates in the progression of COPD. CS has been reported to induce various types of cell death in COPD, including apoptosis, necroptosis, and ferroptosis [4, 5]. Several signaling pathways have been involved in the CS-induced lung epithelial cell death. However, the molecular mechanisms of CS-induced airway epithelial cell death in the pathogenesis of COPD remain to be investigated.

Protein arginine methyltransferases (PRMTs) are a group of enzymes that catalyze the addition of methyl group(s) to nitrogen atoms of guanidinium side chains within arginine residues of proteins that have been exclusively involved in a range of biological processes [6, 7, 8, 9]. PRMT6 is a type I PRMT, which primarily locates in the nucleus and catalyzes asymmetric di-methylation on histone H3 Arginine 2 residue (H3R2me2a) [10]. PRMT6-mediated histone H3R2 methylation (H3R2me2a) generally results in transcriptional repression, possibly via impairment of the transcription activating effect of H3 Lysine 4 trimethylation (H3K4me3) [11, 12]. In addition to its epigenetic function, PRMT6 can also methylate non-histone proteins to modulate a variety of life processes, including protein-protein interactions and gene transcription. PRMT6 methylates p53, p21, and p16, involving in regulation of cell proliferation, cell-cycle arrest, senescence, and apoptosis [13, 14, 15]. PRMT6 knockout mice do not show any particular characteristic phenotypic features; however, its ablation negatively regulates p53, and a gain-of-function mouse model showed that PRMT6 co-activates NF-κB [13, 16]. Strikingly, PRMT6 is an important regulatory factor for cell fate decision. In early zebrafish development, loss function of PRMT6 results in early epiboly defects and apoptosis through activating p38/JNK signaling [17]. PRMT6 protein is aberrantly overexpressed in several cancers including breast, prostate, and lung cancer, and its high level predicts better prognosis [14, 18, 19]. PRMT6 catalyzes CRAF methylation to decrease its RAS binding and alter its downstream MEK/ERK signaling to inhibit the cancer stemness activities of hepatocellular carcinoma [20]. Collectively, these studies implicate the involvement of PRMT6 in the fate decision of multiple cells, but its exact role and underlying mechanisms in the CS-induced airway epithelial cell death remain unclear.

PI3K/AKT signaling pathway is a major player in diverse pathological settings, that include cell death and survival, proliferation, angiogenesis, immune cell activation, and neurological disorders [21]. Phosphorylation at both Thr308 and Ser473 are required for full activation of AKT [22]. PI3K p110 catalytic subunit (PI3Kp110) recruits downstream 3-Phosphoinositide Dependent Protein Kinase 1 (PDK1) to phosphorylate AKT at Thr308 and Mechanistic Target of Rapamycin Kinase 2 (mTORC2) to catalyze Ser473 phosphorylation [23]. On the other hand, several phosphatases have been involved in the regulation of AKT phosphorylation, including Phosphatase And Tensin Homolog (PTEN), PH Domain And Leucine Rich Repeat Protein Phosphatase 1, 2 (PHLPP1, 2), Protein phosphatase 2A catalytic (PPP2AC) and Inositol Polyphosphate-4-Phosphatase Type II B (INPP4b) [24–27]. Thus, stringent regulation of AKT phosphorylation is essential for cell death and survival at physiological scenarios. AKT was reported to be repressed by cigarette smoke-related cellular and animal models [28]. Contradictorily, the inhibitory factor PTEN is believed to be downregulated to cause inflammation via AKT pathway [29, 30]. How PI3K/AKT signals are schematically reprogrammed in cigarette smoke-induced pathophysiological settings needs further dissection.

Our study provides new mechanistic insights showing how CS inhibits PRMT6 to downregulate PI3K/AKT signaling leading to lung epithelial cell death, that provides a potential therapeutic target for COPD.

## RESULTS

### PRMT6 is downregulated in the lung tissue of cigarette smoke treated mice

**Figure 1:**
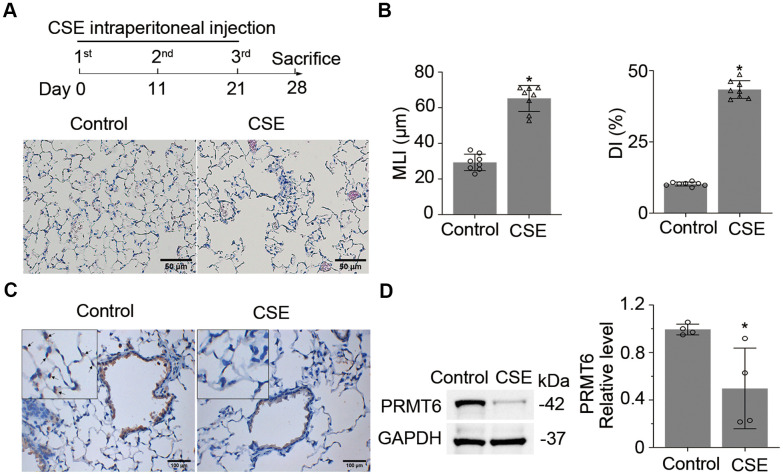
**Cigarette smoke down-regulates PRMT6 in mice lung tissues.** (**A**) Balb/C mice were treated with CSE by intraperitoneal injection for three times in 1 month as described in materials and methods. Lung tissues were stained with H&E, and the emphysematous change of lung tissue was presented in CSE treated group as compared to control mice. (**B**) Mean linear intercept (MLI) and the destructive index (DI) were measured and plotted to show the histological destruction in lung tissues. (**C**) Representative staining of PRMT6 in airway epithelia and alveoli with immunohistochemistry (IHC) analysis. (**D**) Immunoblots of PRMT6 protein in mice lung tissue with GAPDH used as a loading control. Relative protein expression was measured by Image J and shown as mean ± SD. “*” denotes *p* < .05, based on Student t-test.

To explore the role of PRMT6 in cigarette smoke mediated emphysema, we first established an experimental mouse emphysema model by intraperitoneal injection of CSE (Figure 1A, upper panel). Mouse lung histological results show that CSE treatment successfully induced emphysema (Figure 1A, lower panels). Results from MLI and DI analysis evidenced severe morphological destruction of lung tissues in CSE-treated mice (Figure 1B). We then carried out immuno-histochemical (IHC) staining and observed that PRMT6 protein was significantly reduced in the airway epithelia of cigarette smoke-treated mice in comparison with control animals (Figure 1C). Low expression of PRMT6 protein was observed in epithelial cells at both bronchioles and alveoli. Consistent with the results of IHC, immunoblotting results verified the reduced expression of PRMT6 in CSE-induced emphysema lung tissue homogenates (Figure 1D). In all, these results indicate that intraperitoneal injection of CSE successfully induced lung emphysema and PRMT6 protein was downregulated in CSE-induced emphysema mouse lung tissues.

### Cigarette smoke reduces the mRNA and protein expression of PRMT6 in airway epithelial cells

**Figure 2:**
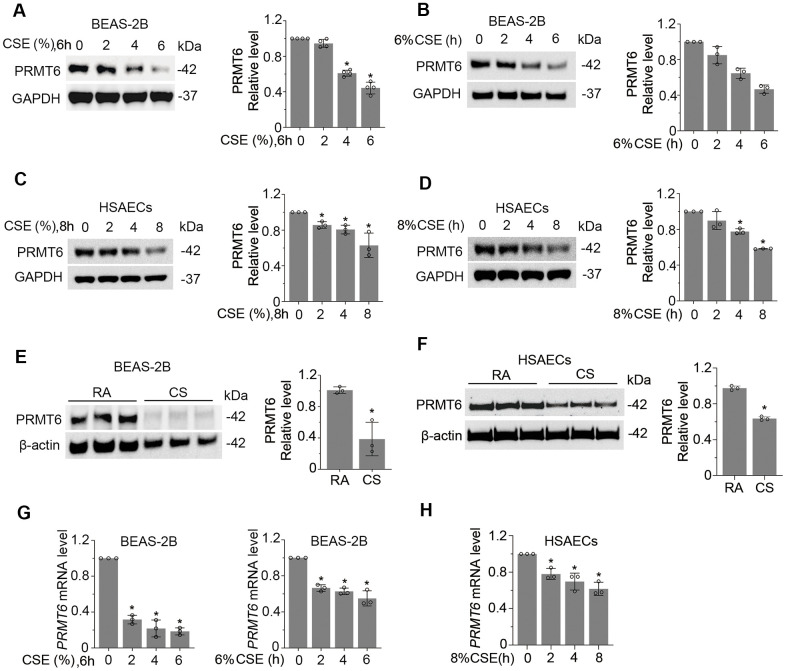
**Cigarette smoke reduces the mRNA and protein expression of PRMT6 in airway epithelial cells.** (**A**, **B**) BEAS-2B cells were treated with CSE in a range of concentrations (**A**) and time points (**B**) as indicated. Cell lysates were subjected to immunoblotting for PRMT6 and GAPDH. The densitometric results were plotted in the right panels. (**C**, **D**) Human primary small airway epithelial cells (HSAECs) were treated with CSE in different concentrations (**C**) for 0, 2, 4, 8h (**D**). Immunoblotting was performed to examine PRMT6 expression. Right panels showed the densitometric results of the blots. (**E**) BEAS-2B cells were exposed to cigarette smoke. Cell lysates were analyzed with PRMT6 and GAPDH via immunoblotting. RA: room air; CS: cigarette smoke. The densitometry results of the blots were plotted in the right panel. (**F**) HSAECs cells were exposed with room air (RA) or cigarette smoke (CS). Cell lysate were obtained and analyzed with PRMT6 and GAPDH immunoblotting. Right panels were the plotted densitometric results. (**G**) Total RNA was extracted from control and CSE-treated BEAS-2B cells (2%, 4%,6% for 6h, and 6% for 0, 2h, 4h, 6h). PRMT6 and GAPDH mRNA levels were determined with qRT-PCR. (**H**) HSAECs were treated with 8% CSE for 2h, 4h and 8h. Total RNA was extracted and applied to qRT-PCR to detect *PRMT6* and *GAPDH* mRNA level. Relative *PRMT6* mRNA level was plotted. Values represent mean ± SD and “*” denotes *p* < .05. Results were representative of at least *n*=3 experiments.

Given the substantial low expression of PRMT6 in CSE-induced emphysema mouse lung tissues, we evaluated the expression of PRMT6 in human bronchial epithelial cells. We first examined PRMT6 expression in BEAS-2B cell lines. PRMT6 protein levels were decreased after CSE treatment in both a concentration and time-dependent manner (Figure 2A, 2B). CSE treatment for 4h at a concentration of 4% was enough to reduce PRMT6 at protein level. Similarly, in primary human small airway epithelial cells treated with CSE expression of PRMT6 was significantly decreased by concentration (Figure 2C), and time (Figure 2D) courses. We next exposed BEAS-2B cells that were grown on transwell inserts as monolayers then taken to air-liquid interface, to assess direct CS or air exposure. Direct CS exposure severely reduced the protein expression of PRMT6 in BEAS-2B cells (Figure 2E). We observed similar results in HSAECs, cigarette smoke exposure reduced PRMT6 protein expression as well (Figure 2F). To understand if CSE impairs PRMT6 expression at transcriptional level, we isolated RNA from CSE-treated BEAS-2B and HSAECs cells and conducted quantitative RT-PCR (qRT-PCR). Results from qRT-PCR showed that CSE reduced *PRMT6* mRNA levels in both concentration and time courses in BEAS-2B cells (Figure 2G). The similar result was also found in HSAECs treated with CSE (Figure 2H). These data indicate that CS reduced PRMT6 protein levels at lung epithelial cells possibly via inhibition of *PRMT6* mRNA expression.

### Genetic depletion of PRMT6 results in AKT dephosphorylation via impairment of PI3K/AKT signaling

**Figure 3:**
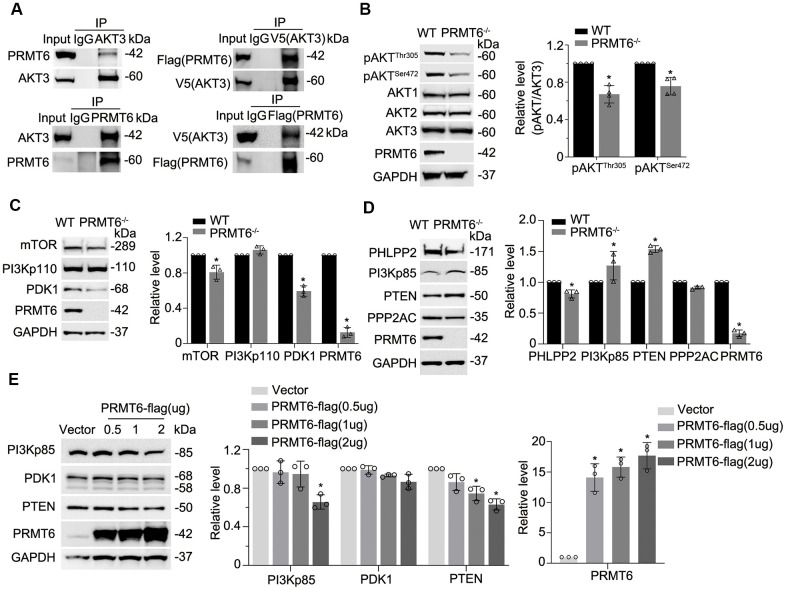
**Genetic depletion of PRMT6 results in AKT dephosphorylation via impairment of PI3K-AKT signaling.** (**A**) BEAS-2B cell lysates were immunoprecipitated with PRMT6 or AKT3 antibody, and the immunoprecipitates were analyzed with AKT3 and PRMT6 immunoblotting as indicated (left two panels). *pcDNA3.1D-PRMT6-FLAG and pcDNA3.1D-AKT3-V5 plasmids* were co-transfected into BEAS-2B cells. After 48h of transfection, cell lysates were immunoprecipitated with FLAG or V5 antibody, and the immunoprecipitants were analyzed with V5 and FLAG immunoblotting as indicated (right two panels). (**B**) PRMT6 CRISPR/Cas9 KO plasmid and HDR plasmid were applied to establish stable PRMT6 gene knockout BEAS-2B cell line. The knockout efficiency was determined by immunoblotting. The cell lysates of wild type (WT) and PRMT6 stable knockout BEAS-2B cell (PRMT6^-/-^) were applied for pAKT^Thr305^, pAKT^Ser472^, AKT isoforms 1, 2, 3 and PRMT6 immunoblotting (left panel). Plotted densitometry results of the pAKT^Thr305^ and pAKT^Ser472^ in WT and PRMT6 knockout group were presented (right panel). (**C,** **D**), Cell lysates of WT and PRMT6^-/-^ BEAS-2B cells were collected and immunoblotted with indicated antibodies. At the right panel of each figure, the plotted densitometry results were presented. (**E**) *pcDNA3.1D-His-V5* control plasmid (Vector) and *pcDNA3.1D-PRMT6-V5 plasmids* were delivered into BEAS-2B cells via electroporation. After 48h, the cell lysates were collected and immunoblotted with indicated antibodies. The plotted densitometry of PI3Kp85, PDK1, PTEN and PRMT6 were presented (middle and right panel). Values represent mean ± SD and “*” denotes *p* < 0.05. Results were representative of at least *n*=3 experiments.

Since PRMT6 is a protein arginine methyltransferase, we presumed that PRMT6 interacts with and post-translationally modifies important molecules that are crucial in leading lung epithelial cell death. Data bank search revealed that PRMT6 interacts with AKT3 (an isoform of AKT) in a recent mass-spectrometric study [31]. Results from immunoprecipitation studies showed that endogenous PRMT6 did associate with AKT3 (Figure 3A, left two panels). We further co-transfected PRMT6-FLAG and AKT3-V5 plasmids into BEAS-2B cell, results also showed that PRMT6 and AKT3 interacted with each other (Figure 3A, right two panels). Unexpectedly, PRMT6 did not change arginine methylation status in AKT3 (data not shown). To linearize the potential biological significance of PRMT6/AKT interaction, we established stable PRMT6-knockout (PRMT6^-/-^) cell lines in BEAS-2B cells using CRISPR/Cas9 knockout techniques. Immunoblotting results showed that PRMT6 protein was completely depleted in PRMT6^-/-^ cells (Figure 3B). Interestingly, KO of PRMT6 substantially inhibited AKT phosphorylation both in pAKT^Thr305^ and pAKT^Ser472^ but did not influence the individual AKT isoform at protein level (Figure 3B) in BEAS-2B cells. These studies suggest that a protein arginine methyltransferase PRMT6 indirectly impairs AKT phosphorylation.

PI3 kinase and downstream signal transduction cascade determines phosphorylation levels of AKT. To better understand PRMT6-mediated molecular pathways in BEAS-2B cells, we conducted an analysis of PI3K/AKT signaling pathways to characterize PRMT6-regulated AKT signaling. Proteins involved in PI3K/AKT signaling pathway were subjected to immunoblotting analysis in PRMT6^-/-^ BEAS-2B cells. Depletion of PRMT6 did not obviously affect PI3Kp110 at protein level, but it decreased downstream kinases mTOR and PDK1 at protein level (Figure 3C). On the other hand, the protein levels of phosphatase PTEN were identified to be substantially increased in PRMT6 deleted cells with respect to WT control cells, an increase of the negative regulatory subunit p85 of PI3K was also noted (Figure 3D, lower panels). Deletion of PRMT6 decreased protein levels of phosphatase PHLPP2 (Figure 3D). Besides, ectopic expression of PRMT6 in BEAS-2B cells reduced PI3K negative regulator p85(PI3Kp85) and PTEN protein levels (Figure 3E). In all, these data suggested that PRMT6 indirectly affect AKT phosphorylation by impairing signal transduction between PI3K and AKT via phosphatase PTEN and PI3Kp85.

### CSE downregulates PI3K/AKT signal transduction in lung epithelial cells

**Figure 4:**
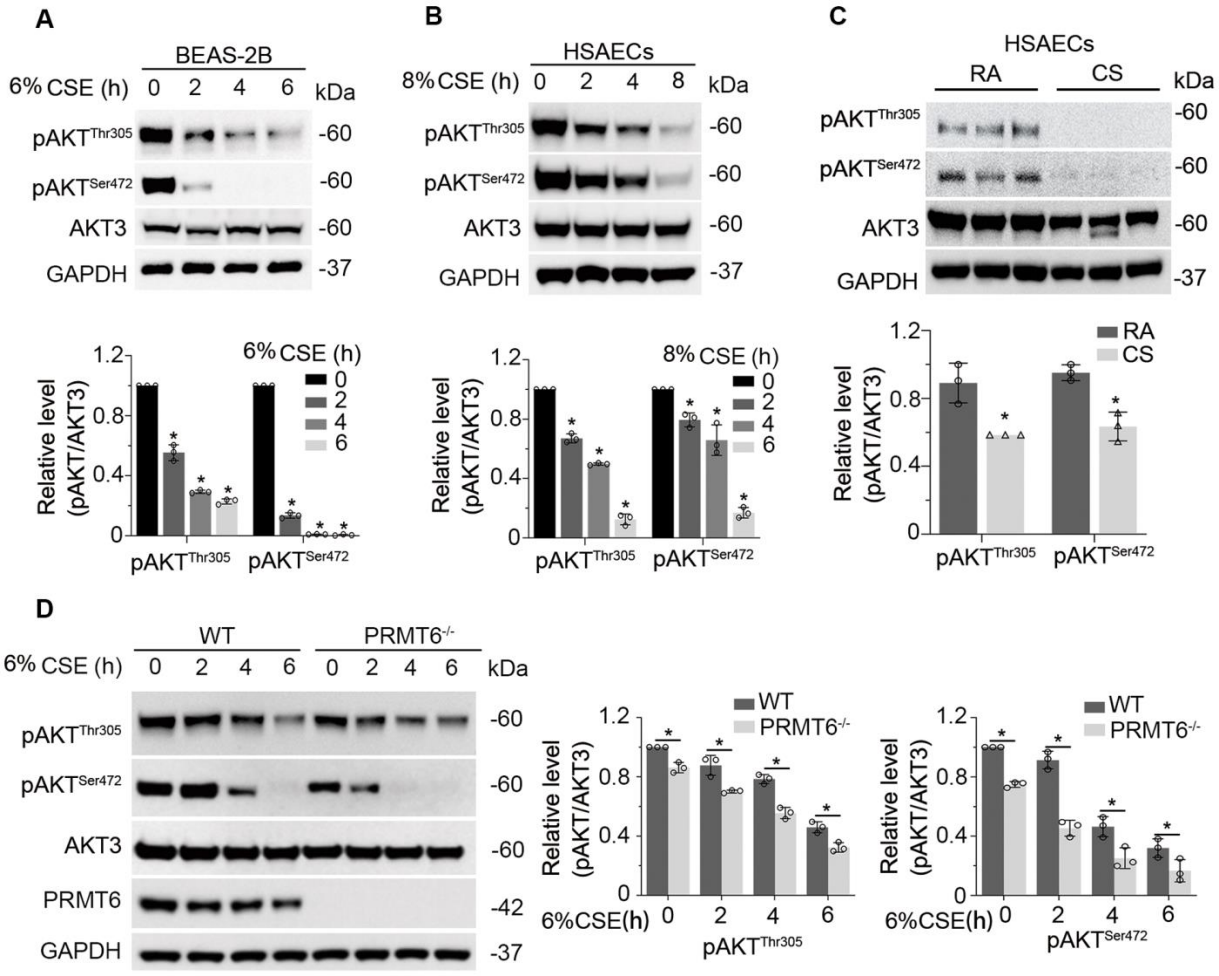
**CSE downregulates PI3K/AKT signal transduction in lung epithelial cells.** (**A**) BEAS-2B cells were treated with 6% CSE for 0, 2, 4, 6h. Cell lysates were immunoblotted with phosphorylated AKT and total AKT. The densitometry results of the blots were presented in the lower panel. (**B**) HSAECs cells were treated with 8% CSE in a range of concentrations as indicated for 0, 2, 4, 8h. Phosphorylated AKT and AKT3 were immunoblotted. The densitometry of the blots was presented in its lower panel. (**C**) HSAECs were applied to room air or cigarette smoke exposure. RA: room air; CS: cigarette smoke. Cell lysates were immunoblotted with pAKT^Thr305^, pAKT^Ser472^, AKT3 and GAPDH antibodies. The densitometry results of the blots were presented in the lower panel. (**D**) Both WT and PRMT6^-/-^ BEAS-2B cells were treated with CSE at different time courses. Cell lysates were subjected to immunoblotting with pAKT^Thr305^, pAKT^Ser472^, AKT3, PRMT6, and GAPDH. Results were shown as mean ± SD and representative of *n*=3 experiments. Statistical significance was indicated as *: *p* < .05.

**Figure 5:**
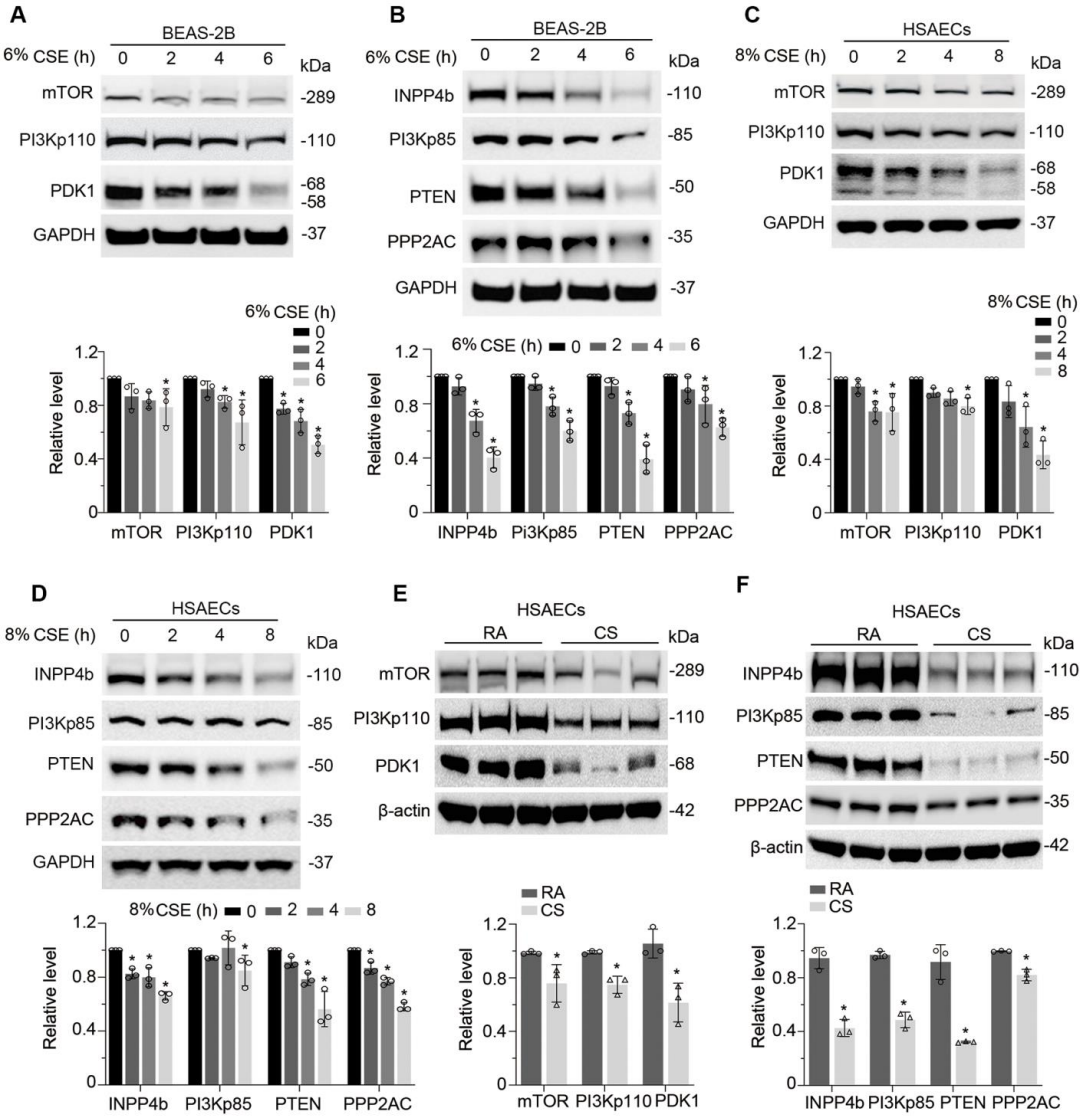
**CSE suppresses PI3K/AKT cascade in epithelial cells.** (**A**, **B**) BEAS-2B cells were treated with CSE at various time points as indicated. Cells lysates were analyzed with mTOR, PI3Kp110, PDK1, INPP4b, PI3Kp85, PTEN, PPP2AC, PRMT6, and GAPDH immunoblotting. The densitometry of the blots was presented in the lower panel. (**C**, **D**) HSAECs cells were treated with CSE at a course of time as indicated. Cells lysates were analyzed with mTOR, PI3Kp110, PDK1, INPP4b, PI3Kp85, PTEN, PPP2AC, PRMT6, and GAPDH immunoblotting. The densitometric results were plotted in the lower panels. (**E**, **F**) HSAECs cells were exposed to room air or cigarette smoke. Cells lysates were collected and analyzed with indicated antibodies immunoblotting. RA: room air; CS: cigarette smoke. The densitometric blots were showed in the lower panel of each figure. Results were representative of *n*=3 experiments. Statistical significance was indicated as *: *p* < .05.

We then studied whether CSE impairs AKT phosphorylation status. As expected, CSE treatment decreased AKT phosphorylation at both sites of Thr305 and Ser472 without obvious effect on the total AKT protein level in BEAS-2B cells (Figure 4A). We observed similar results in HSAECs cells (Figure 4B). *in vitro* cigarette smoke exposure reduced pAKT level in HSAECs as well (Figure 4C). In addition, PRMT6 ablation accelerated the reduction of AKT phosphorylation under CSE treatment (Figure 4D). Since CSE reduced AKT phosphorylation, we were intrigued to determine if CSE treatment impairs PI3K/AKT signaling in airway epithelial cells. Results from immunoblotting analysis showed that CSE decreased the protein levels of PI3K p110 catalytic unit and downstream kinases mTOR and PDK1 (Figure 5A) in BEAS-2B cells. CSE also decreased the protein levels of phosphatase PPP2AC, INPP4b, PTEN, as well as PI3Kp85, the negative regulator of PI3K kinase (Figure 5B). We observed similar results in CSE-treated human small airway epithelial cells (Figure 5C, 5D) and cigarette smoke exposed HSAECs cells (Figure 5E, 5F). Overall, results from these experiments indicated that CSE treatment downregulates most of the molecules in PI3K/ AKT signal transduction.

### CSE suppresses AKT phosphorylation via PRMT6/PTEN signal transduction

**Figure 6:**
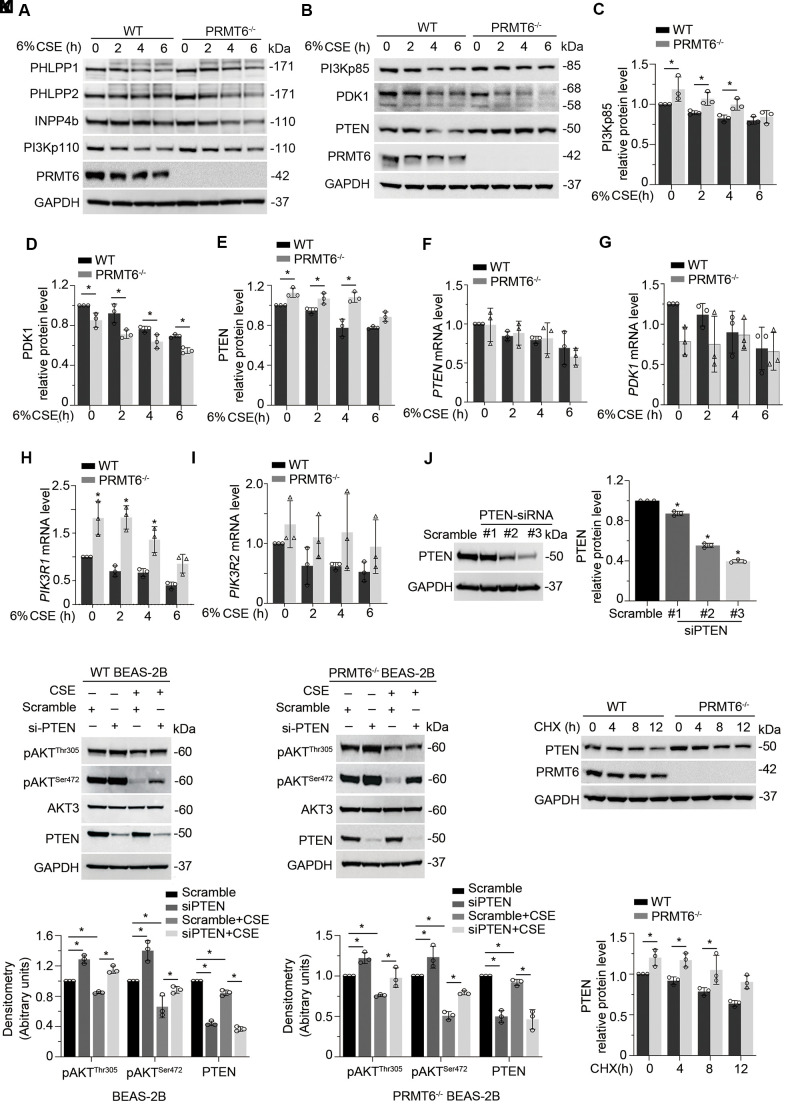
**CSE suppresses AKT phosphorylation via PRMT6/PTEN signal transduction.** (**A**, **B**) WT and PRMT6^-/-^ BEAS-2B cells were treated with CSE at 2, 4, 6h. Western blotting was performed to detect the protein level of PHLPP1, PHLPP2, INPP4b, PI3Kp110, PI3Kp85, PDK1, PTEN, PRMT6 and GAPDH. (**C**–**E**) The plotted densitometry results of PI3Kp85, PDK1 and PTEN protein expression were presented. (**F**–**I**) WT and PRMT6^-/-^ BEAS-2B cells were treated with CSE for 2, 4, 6h. qRT-PCR was performed to detect the mRNA level of *PTEN, PDK1, PIK3R1* and *PIK3R2*. Results of qRT-PCR were shown as mean ± SD and representative of *n*=3 experiments. (**J**) Scramble siRNA and three kinds of double strands PTEN-siRNA were transfected into BEAS-2B separately. PTEN expression was detected by western blotting after transfection of PTEN-siRNA for 72hrs. Plotted PTEN protein level was presented in the right panel. (**K**, **L**) PTEN expression was silenced by DsiRNA in wild type and PRMT6 KO BEAS-2B cells. Western blotting was used to assay the AKT phosphorylation level. Representative blots of PTEN and PRMT6 were shown. (**M**) Cycloheximide (CHX, 100ug/ml) was applied to WT and PRMT6^-/-^ BEAS-2B cells for 0, 4, 8, 12h. Densitometric results of PTEN and PRMT6 blots were plotted (lower panel). Results were representative of *n*=3 experiments. Statistics were measured by 1-way and 2-way ANOVA, *: *p* < 0.05.

To dissect the role of PRMT6 in CSE-mediated inhibition on PI3K/AKT signaling transduction, we treated WT and PRMT6^-/-^ BEAS-2B cells with CSE at different time points. Immunoblotting results showed that ablation of PRMT6 didn’t influence the CSE-decreased protein levels of PHLPP1, PHLPP2, INPP4b and PI3Kp110 (Figure 6A). Notably among the molecules observed, KO of PRMT6 rescued CSE-mediated downregulation of PTEN as well as PI3Kp85 as compared with that in WT BEAS-2B cells, but accelerated CSE-mediated decrease of PDK1 (Figure 6B–6E). PTEN and PI3Kp85 are reported as key negative regulators in AKT phosphorylation. PDK1 is a kinase to specifically promote serine 472 phosphorylation of AKT. Since PRMT6 is a negative regulator of gene transcription, we measured the mRNA levels of the genes in CSE treated PRMT6^-/-^ cells. qRT-PCR results showed that CSE did not impair *PTEN* mRNA expression (Figure 6F), but reduced *PDK1* mRNA expression (Figure 6G), and increased the mRNA expression of PI3Kp85 subunit (*PIK3R1* and *PIK3R2)* (Figure 6H, 6I). Interestingly, PRMT6 deficiency itself didn’t influence the mRNA level of *PTEN* (Figure 6F), but decreased the *PDK1* mRNA level (Figure 6G) and increased the mRNA level of PI3Kp85 subunit, *PIK3R1* (Figure 6H). PTEN was depleted with DsiRNA oligos as shown in #2 and #3 *PTEN*-siRNA after 72hrs of transfection (Figure 6J). Silence of PTEN partly reversed pAKT^Thr305^ and pAKT^Ser472^ level in CSE treated BEAS-2B cell and in CSE treated PRMT6^-/-^ cells (Figure 6K, 6L). PTEN was unstable and underwent protein degradation as the depletion of PRMT6 slowed down this process (Figure 6M). Overall, these data suggested that CSE inhibits PRMT6, furthermore, decreased PRMT6 in-turn affects PTEN, PDK1, and PI3Kp85 subunit to lead to lower AKT phosphorylation.

### PRMT6 overexpression protected CSE-driven lung epithelial cell death

**Figure 7:**
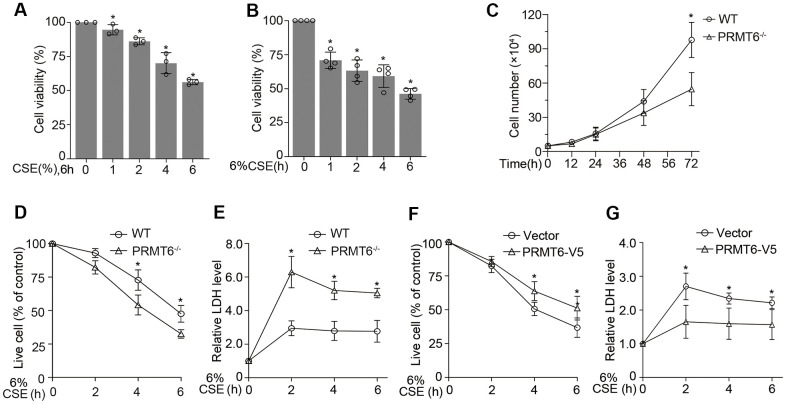
**PRMT6 overexpressed epithelia are protected from CSE driven cell death.** (**A**, **B**) BEAS-2B cells were treated with various concentrations of CSE at indicated time points. Cell viability was measured with MTT assay. (**C**) 5×10^4^ cells of WT or PRMT6^-/-^ cell were cultured for a variety of time points, then stained by trypan blue and counted by TC10 automatic cell counter. (**D**) The trypan blue stained cells were counted by TC10 automatic cell counter. The percentage of live cells relative to control group after CSE treatment in WT and PRMT6^-/-^ groups were presented. (**E**) LDH assay was used to determine cell death induced by CSE treatment in WT and PRMT6^-/-^ groups. Relative LDH activity indicated the epithelial cell death rate were plotted. (**F**) 3 μg of *pcDNA3.1D-PRMT6-V5 plasmid* and control plasmid were transfected into BEAS-2B cells via electroporation. After 48h, the vector and PRMT6 overexpressed BEAS-2B cells were treated with CSE at various time points. The percentage of live cells were counted and compared with control. (**G**) Relative LDH activity under CSE treatment in vector and PRMT6-V5 overexpressed group were determined with LDH assay. Results were shown as mean ± SD and representative of *n*≥3 experiments. Statistics were measured by 1-way and 2-way ANOVA or Student t test, *p < .05 indicated the statistical significance.

Epithelial cell death is a pivotal factor in cigarette smoke-induced airway diseases. CSE decreased cell viability in both concentration and times courses (Figure 7A, 7B). To understand the effect of PRMT6 on cell viability in cigarette smoke exposure, we treated PRMT6^-/-^ cells with CSE at different time points. Deletion of PRMT6 itself reduced cell proliferation (Figure 7C). Furthermore, CSE remarkably decreased cell viability in PRMT6^-/-^ cells compared to WT cells (Figure 7D). Results from LDH assay confirmed that CSE treatment induced more toxic effect in PRMT6 deficient cells (Figure 7E). Furthermore, ectopic overexpression of PRMT6 increased cell viability under CSE treatment (Figure 7F) and reduced CSE-mediated toxicity in the cells (Figure 7G). These results indicate that PRMT6 could functionally improve the ability of epithelial cells to resist on CSE toxicity and alleviate CSE-induced epithelial cell death.

## DISCUSSION

**Figure 8:**
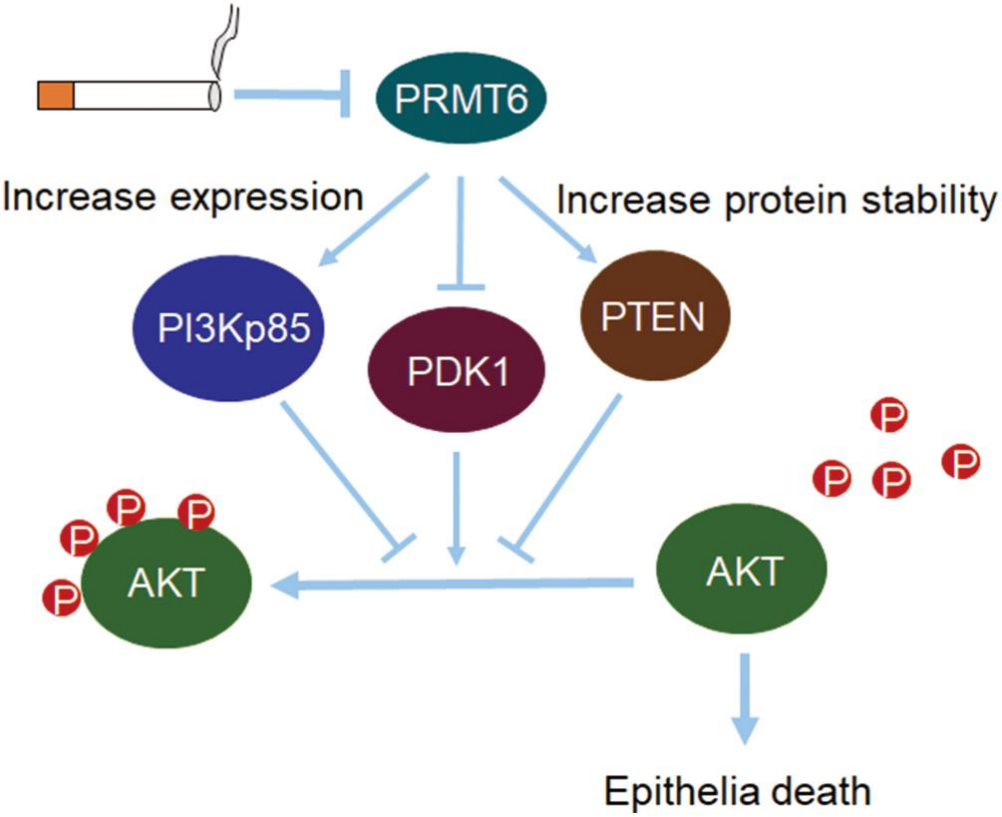
**PRMT6-pAKT axis is crucial in CSE-induced epithelia death.** Cigarette smoke reduces PRMT6 at both mRNA and protein levels. Decreased PRMT6 promotes PI3Kp85 expression and increases the protein stability of PTEN. Changes of PI3Kp85 and PTEN, as well as decreased PDK1, reduce the total phosphorylation level of AKT. Lower level of AKT phosphorylation in turn promotes cigarette smoke-induced lung epithelial cell death.

Our central findings in this study are that, (i) cigarette smoke decreased PRMT6 in cellular and mouse models; (ii) knockout of PRMT6 reduced AKT phosphorylation; (iii) CSE-reduced PRMT6 expression aggravated AKT dephosphorylation via multiple channels in PI3K/AKT signaling; and (iv) PRMT6 deficiency enhanced CSE toxicity in lung epithelial cells (Figure 8). Chronic airway inflammation, tissue destruction, and small airway reduction/lesion are the main pathological features of COPD^1^. Cigarette smoking (CS) is regarded as the most important risk factor for airway inflammation, emphysema, and COPD development [3]. CS induces cell death, increases excessive generation of reactive oxygen species (ROS) and release of inflammatory mediators, and lastly contributes to impaired tissue repair [32]. Furthermore, long-term CS exposure augmented these pathological changes [33]. An overwhelming fact is that the diversified molecular mechanisms of CSE-mediated respiratory pathogenesis and the chemical composition in cigarette smoke may complicate the situation even more. In this study, we added a new paradigm for identification that a histone-modifying enzyme PRMT6 plays a crucial role in CSE-mediated cell death in cellular and rodent models. Several articles reported that PRMT6 aberrantly increased in many cancers like breast, prostate, gastric and lung cancer [14, 18, 19, 34]. Viral infection and LPS also upregulated the protein levels of PRMT6 [35, 36]. However, PRMT6 protein levels are reduced in both CSE cellular and rodent models possibly via gene transcription. PRMT6 was also reported to be decreased in cigarette smokers and in cigarette smoking COPD patients [37]. These findings confirmed the reduction of PRMT6 in COPD and revealed the potential effect of PRMT6 in the pathogenic development of CS-induced COPD.

Our previous observations suggest that PRMT6 plays a protective role in CSE-induced lung damage in mouse models [37]. Besides, PRMT6 might alleviate CSE-induced apoptosis and inflammation in human umbilical vein endothelial cells (HUVECs) by regulating histone methylation [38]. In this study, we identify that PRMT6 modulates a major cell survival and death molecule AKT to cause cell death. Transcriptome analysis of mammary epithelial cells in bigenic NIC-PRMT1Tg and NIC-PRMT6Tg mice revealed deregulated PI3K/AKT pathway, PCR also validated higher mRNA expression of PI3K regulatory subunit 3 (PIK3R3) [39]. Although PRMT6 interacts with AKT, it interestingly does not directly alter the methylation status of AKT. Instead, PRMT6 regulates PDK1 and PI3Kp85 at gene transcription level, and PTEN at protein level. The depletion of PRMT6 increased PTEN stability. Consistent with our findings, a recent study reported that PRMT6 methylate PTEN at Arg159 to repress AKT signaling, and Arg159 mutation within PTEN contributes to cancer development. However, we identified that CSE downregulates both expression of PRMT6 and phosphorylation level of AKT. Among the multiple channels that regulate AKT phosphorylation, *PTEN* has been reported to be important in the pathogenesis of COPD in a SNAP study [40]. Consistent with this report, our results suggest PTEN may contribute to AKT dephosphorylation, which leads to epithelial cell death in experimental CSE-mediated emphysema. Though CSE downregulates PTEN as well, downregulation of PRMT6 may slow down CSE-mediated decrease of PTEN.

Accumulated evidence suggested that histone modifiers are associated with the pathogenesis of COPD and COPD exacerbation [37, 41–46]. Complete loss of NAD-dependent protein deacetylase sirtuin-1(Sirt1) in airway epithelium leads to early development of emphysematous change in mice lungs [46]. Reduction of histone deacetylase 2 (HDAC2) activity and expression in peripheral lung and alveolar macrophages results in amplification of the inflammatory response in COPD [47]. Coactivator- associated arginine methyltransferase-1 (CARM1) haploinsufficiency impairs the function of alveolar epithelial cells, inducing alveolar senescence to promote premature lung aging [43, 44]. Cigarette smoke, as the biggest risk factor of COPD development, may elicit exclusive histone posttranslational modification to impair lung cell dysfunction, cell senescence, and cell death at different time points and dose [4, 43, 44, 48–51]. However, the role of individual histone modifiers in cigarette smoke-induced pathogenesis of COPD may be distinct and needs to be further dissected. Individual histone modifiers may contribute to COPD pathogenesis with distinct mechanisms. For example, CS promotes HDAC6 phosphorylation to disrupt lung endothelial barrier integrity possibly via AKT inactivation [52]. In the present study, we add another paradigm that CSE-induced epithelial death via PRMT6/AKT signaling. The work of understanding the individual histone modifiers in the COPD pathogenesis is ongoing.

## MATERIALS AND METHODS

### Cell culture and reagents

The human bronchial airway epithelial cells (BEAS-2B) and the primary human small airway epithelial cells (HSAECs) were purchased from the American Type Culture Collection (ATCC). BEAS-2B cells were maintained with HITES medium (500 mL DMEM/F12, 2.5 mg insulin, 2.5 mg transferrin, 2.5 mg sodium selenite, 2.5 mg transferrin, 10 μM hydrocortisone, 10 μM β-estradiol, 10 mM HEPES and 2 mM L-glutamine) containing 10% fetal bovine serum (FBS) as previously described [53]. The HSAEC cells were maintained with medium containing 500 mL Airway Cell Basal Medium, 500 μg/mL HSA, 0.6 μM Linoleic Acid, 0.6 μg/mL Lecithin, 6 mM L-Glutamine, 0.4% Extract P, 1.0 μM Epinephrine, 5 μg/mL Transferrin, 10 nM T3, 1 μg/mL Hydrocortisone, rh EGF 5 ng/mL, and 5 μg/mL rh Insulin (Bronchial Epithelial Growth Kit, Cat#: ATCC PCS-300-040). The V5 antibody (Cat#: 46-0705), pcDNA3.1D-His-V5-TOPO cloning kit (Cat#: K490001) and *Escherichia coli* Top10 competent cells (Cat#: C404006) were from Invitrogen (St. Louis, MO, USA). Antibodies for PRMT6 (Cat#:1461s), AKT1(Cat#: 2938s), AKT2 (Cat#:3063s), AKT3 (Cat#:14982s), mTOR (Cat#:2983s), INPP4b (Cat#: 8450s), PI3Kp85 (Cat#:4257s), and PDK1 (Cat#: 3062s) were from Cell Signaling (Danvers, MA, USA). pAKT^Thr305^ (Cat#: sc-271966), pAKT^Ser472^ (Cat#: sc-81433), PTEN (Cat#: sc-79974), and PI3Kp110 (Cat#: sc-8010) were from Santa Cruz Biotechnology (Dallas, TX, USA). PHLPP2 (Cat#: A300-661A) antibodies were from Bethyl Laboratories (Montgomery, TX, USA). β-actin (Cat#: A3853) and Flag (Cat#: F1804) antibodies were from Sigma (Carlsbad, CA, USA). Antibodies for PHLPP1 (Cat#: 22789-AP) and GAPDH (Cat#: 60004-1-Ig) were from Proteintech Group (Rosemont, IL, USA). Immobilized protein A/G Agarose beads (Cat#: 20421) were from Pierce (Rockford, IL, USA). All other reagents were of the highest grade available commercially.

### CSE solution preparation and CS exposure

The aqueous CSE was prepared according to a previously described method with a slight modification [54]. Briefly, 10 mL of serum-free sterile DMEM medium was drawn into a 60-mL plastic syringe. Subsequently, 40 mL of one puff of cigarette smoke (filtered 3R4F cigarettes; the Kentucky Tobacco Research and Development Center at the University of Kentucky, Lexington, KY, USA) was drawn into the syringe and mixed with the medium by vigorous shaking for 30s. One cigarette was used for each 10 mL of medium, and 11 puffs were taken from one cigarette. The CSE solution, filtered through an aseptic 0.22-μm filter, was considered as 100%. The aqueous CSE was diluted in culture medium before use, and the CSE solution was prepared by same person with same method and was used within 30 min after preparation in all experiments. For animal experiments, smoke from cigarette was passed through sterile PBS (2 mL per cigarette) using a vacuum pump to prepare the CSE. The CSE mixture was then filtered with a 0.22 μm filter to exclude bacteria and particles.

### In vitro smoke exposure

BEAS-2B cells or HSAECs grown on transwell inserts were exposed to cigarette smoke or room air using a Vitrocell exposure chamber (Vitrocell Systems, Germany), which was used to deliver apical gaseous smoke or air to cells at air-liquid interface. BEAS-2B or HSAECs cells were exposed to 4 cigarette exposures in 32 minutes using an International Organization for Standardization (ISO) protocol. Cell lysates were prepared for western blot analysis.

### Animals experiments

The animal protocol was approved by the Animal Care and Use Committee of the Second Xiangya Hospital of Central-South University. Twenty adult Balb/c mice (21~23 g of weight at ages of 10 weeks) were randomly divided into two groups: control group and CSE group. The CSE group was injected intraperitoneally with 0.3 mL of CSE per mouse on day 0, 11 and 21 as previously described [37], while control mice received PBS only. During the 4-week period of the experiment, all mice were inhabited same special pathogen free (SPF) living environment. On day 28 the mice were sacrificed and lung tissues were processed for histological and immunoblotting analysis.

### Morphological assessment and Immunohistochemistry

Mice lungs were fixed with 10% neutral formalin and 3.5-μm thick paraffin sections were cut. Sections were stained with hematoxylin and eosin (H&E). Emphysematous changes were assessed by measurement of both the mean linear intercept (MLI) and the destructive index (DI) as previously described [55]. Immunostaining was performed using the two-step staining kit (cat#: PV-6002, ZSGB-Bio, Beijing, China). After antigen retrieval in citrate buffer (pH 6.0) for 2 min in an autoclave, 0.3% hydrogen peroxide was applied to the samples for 15 min and then, the sections were incubated with goat serum for 10 min (cat#: 50197Z, Thermo Fisher Scientific, Waltham, MA, USA). The samples were incubated with PRMT6 rabbit polyclonal antibody (dilution, 1:100; Cat#: 15395-1-AP, Proteintech Group, IL, USA) at 4° C overnight. Samples were then incubated with goat anti-rabbit IgG antibody conjugated with peroxidase (dilution, 1:200; Cat#: A0545; Sigma-Aldrich; Merck Millipore, MO, USA) for 10 min at room temperature. The DAB Detection kit (cat#: ZLI-9017, ZSGB-Bio, Beijing, China) was used for staining. Counterstaining with hematoxylin was performed, and the sections were dehydrated in ethanol prior to mounting. Images were acquired by Motic BA210T microscope (Motic, Hongkong, China).

### CRISPR/Cas9 PRMT6 knock-out cell line

PRMT6 CRISPR/Cas9 knockout (KO) plasmids and guiding RNA were designed and constructed by Santa Cruz Biotechnology Inc. BEAS-2B cells were co-transfected with PRMT6 CRISPR/Cas9 KO plasmid (Cat#: sc-403433) and HDR plasmid (Cat#: sc-403433-HDR) using UltraCruz Transfection Reagent (Cat#: sc-395739). PRMT6 stable KO cells were selected by puromycin (Cat#: P9620, Sigma, Carlsbad, CA, USA) at a concentration of 1 μg / mL for 10 days following the instruction from the manufacturer. Single colonies were picked up and PRMT6 KO was confirmed by immunoblotting.

### Plasmid and DsiRNA transfection

All plasmids were introduced into BEAS-2B cells by electroporation, using a nuclear transfection apparatus (Amaxa Biosystems, Gaithersburg, MD, USA) with a preset program T-013 as previously described [53]. Dicer-substrate short interfering RNAs (DsiRNA) against PTEN, including PTEN-DsiRNA-1, PTEN-DsiRNA -2, PTEN-DsiRNA -3 and scrambled-DsiRNA were purchased from Integrated DNA Technology (IDT). *PRMT6* in *pcDNA3.1-C-(k)DYK* plasmid (clone ID: OHu17468) and *AKT3* (clone ID:OHu21214D) were purchased from Genescript (Piscataway, NJ, USA), and was subcloned to *pcDNA3.1D/V5-His* expression vector (Invitrogen, cat#: K4900-40) by PCR. One μg of expression plasmids or 20 pmol DsiRNA constructs were delivered into one million cells in 100 μL of transfection buffer (20 mM HEPES in PBS buffer) by electroporation. After electroporation, the cells were cultured with 2 mL of conditional HITES medium in 6-well plates for 24-72 h for further analyses.

### Immunoblotting and immunoprecipitation

Cells in a 6-well plate were washed with cold PBS and collected in cold 0.1 mL of cell lysis buffer A (1×PBS, 0.5% Triton X-100, and protease inhibitor). Sonication was used to fully break the cells in the lysis buffer, and the cell lysate was cleared with centrifugation at 10,000 g for 10 min at 4° C. Equal amount of cell lysates (20 μg) in SDS-loading dye were subjected to SDS-PAGE and the proteins were transferred to a nitrocellulose membrane. Membranes were blocked with 5% non-fat milk for 1h at room temperature and incubated overnight with the primary antibody (1: 1000) at 4° C. After washing with Tris-buffered saline containing 0.1% Tween 20 (TBS-T) for 3 times, the membranes were incubated with HRP-conjugated secondary antibody (1: 5000-1: 10000). Proteins were visualized using ECL reagents and images were acquired with a Kodak in vivo detection system (Kodak, USA).

For immunoprecipitation, BEAS-2B cells were lysed with an IP lysis buffer (1×PBS, 0.5% Tween 20, and protease inhibitors) as described above. Equal amounts of cell lysates (containing 1 mg of total protein) were incubated with specific primary antibodies overnight at 4° C, followed by additional incubation with 35 μL of protein A/G-agarose beads for 2h. The immunoprecipitants were washed 3 times with 0.5 % Tween 20 in ice-cold phosphate-buffered saline and analyzed by immunoblotting.

### The quantitative reverse transcription polymerase chain reaction (qRT-PCR)

Total cellular RNA was isolated from BEAS-2B cell lines using a RNeasy Mini Kit (Cat#: 74104) purchased from QIAGEN. High-capacity RNA-to-DNA kit (Cat#: 4387406, Applied Biosystems) was applied to synthesize cDNA from 1 μg of total RNA in the reverse transcription reaction. qRT-PCR was performed in a Bio-Rad CFX96 Real-Time PCR system with a cycling program (50° C for 2 min, 95° C for 2 min, followed by 39 cycles of 95° C for 15 sed, 55° C for 15sec, and 72° C for 1 min). The primers used in qRT-PCR were listed below: *PRMT6* forward: 5′-CAAGACACGGACGTTTCAG-3′ and reverse: 5′-CCTGGTCTCCCACTTTGTA -3′, *PDK1* forward: 5′-CCAGTCCAGCGTGGTGTTATG-3′ and reverse: 5′-CCGGTTTAAGGTCCCTGTGAATG-3′, *PIK3R1* forward: 5′-GTGGACCTCATCAATCACTAC-3′ and reverse: 5′-CCAGCCACTCGTTGATTT-3′, *PIK3R2* forward: 5′-CTCTACCCTGTGTCCAAATAC-3′ and reverse: 5′-CTTGTCGATCTCTCTGTTGTC-3′, *PTEN* forward: 5′-CCACCACAGCTAGAACTTATC-3′ and reverse: 5′-GACTCAGTGGTGTCAGAATATC -3′, *GAPDH* forward: 5′-GTATGACAACAGCCTCAAGAT-3′ and reverse: 5′-GTCCTTCCACGATACCAAAG-3′. The relative mRNA level of interest gene was presented as compare to *GAPDH* mRNA [56].

### Cell viability and proliferation assay

The viability of BEAS-2B cells was measured using a 3-(4,5-dimethylthiazol-2-yl)-2,5-diphenyltetrazolium bromide (MTT) assay. BEAS-2B cells were inoculated at a density of 5,000 cells per well in a 96-well plate and cultured for 24h. Cells were cultured with 10 μL MTT solution (5 mg / mL, Cat#: V13154, Life technologies, USA) at 37° C for 4h, then 200 μL of dimethyl sulfoxide was added to dissolve the crystals. Absorbance at 560 nm was read on a SpectraMax M2 microplate reader (Molecular Devices, CA, USA). As a complementary approach, cells were stained with trypan blue, total and alive cell numbers were measured with a TC10 automatic cell counter (Bio-Rad, CA, USA).

### Lactate dehydrogenase (LDH) assay

Cell death was detected by LDH assay. LDH activity in the culture media of the control cells and the CSE-treated cells was determined using LDH kit (Cat. #MK401, Takara, Japan) according to the manufacturer's instructions. Light absorption was measured at wavelength of 490 nm using a SpectraMax M2 microplate reader (Molecular Devices, CA, USA). The relative LDH activity indicated the epithelial cell death and the data were plotted in a bar graph.

### Statistical analysis

All quantified data represent an average of at least three experiments, and the results are presented as the mean ± standard deviation (mean ± SD). Statistical analysis was carried out by GraphPad Prism 5 software (version 8.0) with t student test, one-way analysis of variance (ANOVA) and two-way ANOVA of variance accordingly. *p* values less than 0.05 is considered indicative of statistically significant difference.

## References

[1] Vogelmeier CF, Criner GJ, Martinez FJ, Anzueto A, Barnes PJ, Bourbeau J, Celli BR, Chen R, Decramer M, Fabbri LM, Frith P, Halpin DM, López Varela MV, et al. Global strategy for the diagnosis, management, and prevention of chronic obstructive lung disease 2017 report. Gold executive summary. Am J Respir Crit Care Med. 2017; 195:557–82. 10.1164/rccm.201701-0218PP 28128970

[2] Kang MJ, Lee CG, Lee JY, Dela Cruz CS, Chen ZJ, Enelow R, Elias JA. Cigarette smoke selectively enhances viral PAMP- and virus-induced pulmonary innate immune and remodeling responses in mice. J Clin Invest. 2008; 118:2771–84. 10.1172/JCI32709 18654661 PMC2483678

[3] Agustí A, Hogg JC. Update on the pathogenesis of chronic obstructive pulmonary disease. N Engl J Med. 2019; 381:1248–56. 10.1056/NEJMra1900475 31553836

[4] Mizumura K, Cloonan SM, Nakahira K, Bhashyam AR, Cervo M, Kitada T, Glass K, Owen CA, Mahmood A, Washko GR, Hashimoto S, Ryter SW, Choi AM. Mitophagy-dependent necroptosis contributes to the pathogenesis of COPD. J Clin Invest. 2014; 124:3987–4003. 10.1172/JCI74985 25083992 PMC4151233

[5] Yoshida M, Minagawa S, Araya J, Sakamoto T, Hara H, Tsubouchi K, Hosaka Y, Ichikawa A, Saito N, Kadota T, Sato N, Kurita Y, Kobayashi K, et al. Involvement of cigarette smoke-induced epithelial cell ferroptosis in COPD pathogenesis. Nat Commun. 2019; 10:3145. 10.1038/s41467-019-10991-7 31316058 PMC6637122

[6] Blanc RS, Richard S. Arginine methylation: the coming of age. Mol Cell. 2017; 65:8–24. 10.1016/j.molcel.2016.11.003 28061334

[7] Auclair Y, Richard S. The role of arginine methylation in the DNA damage response. DNA Repair (Amst). 2013; 12:459–65. 10.1016/j.dnarep.2013.04.006 23684798

[8] Yang Y, Bedford MT. Protein arginine methyltransferases and cancer. Nat Rev Cancer. 2013; 13:37–50. 10.1038/nrc3409 23235912

[9] Guccione E, Richard S. The regulation, functions and clinical relevance of arginine methylation. Nat Rev Mol Cell Biol. 2019; 20:642–57. 10.1038/s41580-019-0155-x 31350521

[10] Wu H, Zheng W, Eram MS, Vhuiyan M, Dong A, Zeng H, He H, Brown P, Frankel A, Vedadi M, Luo M, Min J. Structural basis of arginine asymmetrical dimethylation by PRMT6. Biochem J. 2016; 473:3049–63. 10.1042/BCJ20160537 27480107 PMC5280038

[11] Guccione E, Bassi C, Casadio F, Martinato F, Cesaroni M, Schuchlautz H, Lüscher B, Amati B. Methylation of histone H3R2 by PRMT6 and H3K4 by an MLL complex are mutually exclusive. Nature. 2007; 449:933–37. 10.1038/nature06166 17898714

[12] Hyllus D, Stein C, Schnabel K, Schiltz E, Imhof A, Dou Y, Hsieh J, Bauer UM. PRMT6-mediated methylation of R2 in histone H3 antagonizes H3 K4 trimethylation. Genes Dev. 2007; 21:3369–80. 10.1101/gad.447007 18079182 PMC2113036

[13] Neault M, Mallette FA, Vogel G, Michaud-Levesque J, Richard S. Ablation of PRMT6 reveals a role as a negative transcriptional regulator of the p53 tumor suppressor. Nucleic Acids Res. 2012; 40:9513–21. 10.1093/nar/gks764 22904064 PMC3479207

[14] Phalke S, Mzoughi S, Bezzi M, Jennifer N, Mok WC, Low DH, Thike AA, Kuznetsov VA, Tan PH, Voorhoeve PM, Guccione E. P53-independent regulation of p21Waf1/Cip1 expression and senescence by PRMT6. Nucleic Acids Res. 2012; 40:9534–42. 10.1093/nar/gks858 22987071 PMC3479215

[15] Stein C, Riedl S, Rüthnick D, Nötzold RR, Bauer UM. The arginine methyltransferase PRMT6 regulates cell proliferation and senescence through transcriptional repression of tumor suppressor genes. Nucleic Acids Res. 2012; 40:9522–33. 10.1093/nar/gks767 22904088 PMC3479209

[16] Di Lorenzo A, Yang Y, Macaluso M, Bedford MT. A gain-of-function mouse model identifies PRMT6 as a NF-κB coactivator. Nucleic Acids Res. 2014; 42:8297–309. 10.1093/nar/gku530 24939901 PMC4117762

[17] Zhao XX, Zhang YB, Ni PL, Wu ZL, Yan YC, Li YP. Protein arginine methyltransferase 6 (Prmt6) is essential for early zebrafish development through the direct suppression of gadd45αa stress sensor gene. J Biol Chem. 2016; 291:402–12. 10.1074/jbc.M115.666347 26487724 PMC4697175

[18] Almeida-Rios D, Graça I, Vieira FQ, Ramalho-Carvalho J, Pereira-Silva E, Martins AT, Oliveira J, Gonçalves CS, Costa BM, Henrique R, Jerónimo C. Histone methyltransferase PRMT6 plays an oncogenic role of in prostate cancer. Oncotarget. 2016; 7:53018–28. 10.18632/oncotarget.10061 27323813 PMC5288165

[19] Yoshimatsu M, Toyokawa G, Hayami S, Unoki M, Tsunoda T, Field HI, Kelly JD, Neal DE, Maehara Y, Ponder BA, Nakamura Y, Hamamoto R. Dysregulation of PRMT1 and PRMT6, type I arginine methyltransferases, is involved in various types of human cancers. Int J Cancer. 2011; 128:562–73. 10.1002/ijc.25366 20473859

[20] Chan LH, Zhou L, Ng KY, Wong TL, Lee TK, Sharma R, Loong JH, Ching YP, Yuan YF, Xie D, Lo CM, Man K, Artegiani B, et al. PRMT6 regulates RAS/RAF binding and MEK/ERK-mediated cancer stemness activities in hepatocellular carcinoma through CRAF methylation. Cell Rep. 2018; 25:690–701.e8. 10.1016/j.celrep.2018.09.053 30332648

[21] Manning BD, Toker A. AKT/PKB signaling: navigating the network. Cell. 2017; 169:381–405. 10.1016/j.cell.2017.04.001 28431241 PMC5546324

[22] Alessi DR, Caudwell FB, Andjelkovic M, Hemmings BA, Cohen P. Molecular basis for the substrate specificity of protein kinase B; comparison with MAPKAP kinase-1 and p70 S6 kinase. FEBS Lett. 1996; 399:333–38. 10.1016/s0014-5793(96)01370-1 8985174

[23] Sarbassov DD, Guertin DA, Ali SM, Sabatini DM. Phosphorylation and regulation of Akt/PKB by the rictor-mTOR complex. Science. 2005; 307:1098–101. 10.1126/science.1106148 15718470

[24] Gao T, Furnari F, Newton AC. PHLPP: a phosphatase that directly dephosphorylates Akt, promotes apoptosis, and suppresses tumor growth. Mol Cell. 2005; 18:13–24. 10.1016/j.molcel.2005.03.008 15808505

[25] Fedele CG, Ooms LM, Ho M, Vieusseux J, O’Toole SA, Millar EK, Lopez-Knowles E, Sriratana A, Gurung R, Baglietto L, Giles GG, Bailey CG, Rasko JE, et al. Inositol polyphosphate 4-phosphatase II regulates PI3K/Akt signaling and is lost in human basal-like breast cancers. Proc Natl Acad Sci USA. 2010; 107:22231–36. 10.1073/pnas.1015245107 21127264 PMC3009830

[26] Padmanabhan S, Mukhopadhyay A, Narasimhan SD, Tesz G, Czech MP, Tissenbaum HA. A PP2A regulatory subunit regulates C. Elegans insulin/IGF-1 signaling by modulating AKT-1 phosphorylation. Cell. 2009; 136:939–51. 10.1016/j.cell.2009.01.025 19249087 PMC2707143

[27] Malek M, Kielkowska A, Chessa T, Anderson KE, Barneda D, Pir P, Nakanishi H, Eguchi S, Koizumi A, Sasaki J, Juvin V, Kiselev VY, Niewczas I, et al. PTEN regulates PI(3,4)P_2_ signaling downstream of class I PI3K. Mol Cell. 2017; 68:566–80.e10. 10.1016/j.molcel.2017.09.024 29056325 PMC5678281

[28] Kim SY, Kim HJ, Park MK, Huh JW, Park HY, Ha SY, Shin JH, Lee YS. Mitochondrial E3 ubiquitin protein ligase 1 mediates cigarette smoke-induced endothelial cell death and dysfunction. Am J Respir Cell Mol Biol. 2016; 54:284–96. 10.1165/rcmb.2014-0377OC 26203915

[29] Yanagisawa S, Baker JR, Vuppusetty C, Fenwick P, Donnelly LE, Ito K, Barnes PJ. Decreased phosphatase PTEN amplifies PI3K signaling and enhances proinflammatory cytokine release in COPD. Am J Physiol Lung Cell Mol Physiol. 2017; 313:L230–39. 10.1152/ajplung.00382.2016 28522564 PMC5582930

[30] Kamo N, Ke B, Busuttil RW, Kupiec-Weglinski JW. PTEN-mediated Akt/β-catenin/Foxo1 signaling regulates innate immune responses in mouse liver ischemia/reperfusion injury. Hepatology. 2013; 57:289–98. 10.1002/hep.25958 22807038 PMC3524373

[31] Huttlin EL, Bruckner RJ, Paulo JA, Cannon JR, Ting L, Baltier K, Colby G, Gebreab F, Gygi MP, Parzen H, Szpyt J, Tam S, Zarraga G, et al. Architecture of the human interactome defines protein communities and disease networks. Nature. 2017; 545:505–09. 10.1038/nature22366 28514442 PMC5531611

[32] Yao H, Rahman I. Current concepts on oxidative/carbonyl stress, inflammation and epigenetics in pathogenesis of chronic obstructive pulmonary disease. Toxicol Appl Pharmacol. 2011; 254:72–85. 10.1016/j.taap.2009.10.022 21296096 PMC3107364

[33] Tzortzaki EG, Papi A, Neofytou E, Soulitzis N, Siafakas NM. Immune and genetic mechanisms in COPD: possible targets for therapeutic interventions. Curr Drug Targets. 2013; 14:141–48. 10.2174/1389450111314020002 23256714

[34] Okuno K, Akiyama Y, Shimada S, Nakagawa M, Tanioka T, Inokuchi M, Yamaoka S, Kojima K, Tanaka S. Asymmetric dimethylation at histone H3 arginine 2 by PRMT6 in gastric cancer progression. Carcinogenesis. 2019; 40:15–26. 10.1093/carcin/bgy147 30508037

[35] Tsai KD, Lee WX, Chen W, Chen BY, Chen KL, Hsiao TC, Wang SH, Lee YJ, Liang SY, Shieh JC, Lin TH. Upregulation of PRMT6 by LPS suppresses klotho expression through interaction with NF-κB in glomerular mesangial cells. J Cell Biochem. 2018; 119:3404–16. 10.1002/jcb.26511 29131380

[36] Zhang H, Han C, Li T, Li N, Cao X. The methyltransferase PRMT6 attenuates antiviral innate immunity by blocking TBK1-IRF3 signaling. Cell Mol Immunol. 2019; 16:800–809. 29973649 10.1038/s41423-018-0057-4 PMC6804946

[37] He X, Li T, Kang N, Zeng H, Ren S, Zong D, Li J, Cai S, Chen P, Chen Y. The protective effect of PRMT6 overexpression on cigarette smoke extract-induced murine emphysema model. Int J Chron Obstruct Pulmon Dis. 2017; 12:3245–54. 10.2147/COPD.S144881 29138553 PMC5680965

[38] Kang N, Chen P, Chen Y, Zeng H, He X, Zhu Y. PRMT6 mediates CSE induced inflammation and apoptosis. Int Immunopharmacol. 2015; 24:95–101. 10.1016/j.intimp.2014.10.029 25481537

[39] Watson ZL, Bitler BG. Type I protein arginine methyltransferases overexpression promotes transformation and potentiates Her2/Neu-driven tumorigenesis. Cancer Res. 2019; 79:3–4. 10.1158/0008-5472.CAN-18-3552 30602621

[40] Hosgood HD 3rd, Menashe I, He X, Chanock S, Lan Q. PTEN identified as important risk factor of chronic obstructive pulmonary disease. Respir Med. 2009; 103:1866–70. 10.1016/j.rmed.2009.06.016 19625176 PMC2783799

[41] Barnes PJ. Corticosteroid resistance in patients with asthma and chronic obstructive pulmonary disease. J Allergy Clin Immunol. 2013; 131:636–45. 10.1016/j.jaci.2012.12.1564 23360759

[42] Milara J, Lluch J, Almudever P, Freire J, Xiaozhong Q, Cortijo J. Roflumilast N-oxide reverses corticosteroid resistance in neutrophils from patients with chronic obstructive pulmonary disease. J Allergy Clin Immunol. 2014; 134:314–22. 10.1016/j.jaci.2014.02.001 24636089

[43] Sarker RS, Conlon TM, Morrone C, Srivastava B, Konyalilar N, Verleden SE, Bayram H, Fehrenbach H, Yildirim AÖ. CARM1 regulates senescence during airway epithelial cell injury in COPD pathogenesis. Am J Physiol Lung Cell Mol Physiol. 2019; 317:L602–14. 10.1152/ajplung.00441.2018 31461302

[44] Sarker RS, John-Schuster G, Bohla A, Mutze K, Burgstaller G, Bedford MT, Königshoff M, Eickelberg O, Yildirim AÖ. Coactivator-associated arginine methyltransferase-1 function in alveolar epithelial senescence and elastase-induced emphysema susceptibility. Am J Respir Cell Mol Biol. 2015; 53:769–81. 10.1165/rcmb.2014-0216OC 25906418 PMC5455466

[45] Zakrzewicz D, Zakrzewicz A, Preissner KT, Markart P, Wygrecka M. Protein arginine methyltransferases (PRMTs): promising targets for the treatment of pulmonary disorders. Int J Mol Sci. 2012; 13:12383–400. 10.3390/ijms131012383 23202904 PMC3497278

[46] Yao H, Chung S, Hwang JW, Rajendrasozhan S, Sundar IK, Dean DA, McBurney MW, Guarente L, Gu W, Rönty M, Kinnula VL, Rahman I. SIRT1 protects against emphysema via FOXO3-mediated reduction of premature senescence in mice. J Clin Invest. 2012; 122:2032–45. 10.1172/JCI60132 22546858 PMC3366403

[47] Barnes PJ. Role of HDAC2 in the pathophysiology of COPD. Annu Rev Physiol. 2009; 71:451–64. 10.1146/annurev.physiol.010908.163257 18817512

[48] Messner B, Frotschnig S, Steinacher-Nigisch A, Winter B, Eichmair E, Gebetsberger J, Schwaiger S, Ploner C, Laufer G, Bernhard D. Apoptosis and necrosis: two different outcomes of cigarette smoke condensate-induced endothelial cell death. Cell Death Dis. 2012; 3:e424. 10.1038/cddis.2012.162 23152060 PMC3542598

[49] Sauler M, Bazan IS, Lee PJ. Cell death in the lung: the apoptosis-necroptosis axis. Annu Rev Physiol. 2019; 81:375–402. 10.1146/annurev-physiol-020518-114320 30485762 PMC6598441

[50] Ito S, Araya J, Kurita Y, Kobayashi K, Takasaka N, Yoshida M, Hara H, Minagawa S, Wakui H, Fujii S, Kojima J, Shimizu K, Numata T, et al. PARK2-mediated mitophagy is involved in regulation of HBEC senescence in COPD pathogenesis. Autophagy. 2015; 11:547–59. 10.1080/15548627.2015.1017190 25714760 PMC4502689

[51] Sundar IK, Nevid MZ, Friedman AE, Rahman I. Cigarette smoke induces distinct histone modifications in lung cells: implications for the pathogenesis of COPD and lung cancer. J Proteome Res. 2014; 13:982–96. 10.1021/pr400998n 24283195 PMC3975679

[52] Borgas D, Chambers E, Newton J, Ko J, Rivera S, Rounds S, Lu Q. Cigarette smoke disrupted lung endothelial barrier integrity and increased susceptibility to acute lung injury via histone deacetylase 6. Am J Respir Cell Mol Biol. 2016; 54:683–96. 10.1165/rcmb.2015-0149OC 26452072 PMC4942194

[53] Lai Y, Li J, Li X, Zou C. Lipopolysaccharide modulates p300 and Sirt1 to promote PRMT1 stability via an SCF^Fbxl17^-recognized acetyldegron. J Cell Sci. 2017; 130:3578–87. 10.1242/jcs.206904 28883095 PMC5665448

[54] Long C, Lai Y, Li T, Nyunoya T, Zou C. Cigarette smoke extract modulates Pseudomonas aeruginosa bacterial load via USP25/HDAC11 axis in lung epithelial cells. Am J Physiol Lung Cell Mol Physiol. 2020; 318:L252–63. 10.1152/ajplung.00142.2019 31746627 PMC7034647

[55] Chen Y, Hanaoka M, Chen P, Droma Y, Voelkel NF, Kubo K. Protective effect of beraprost sodium, a stable prostacyclin analog, in the development of cigarette smoke extract-induced emphysema. Am J Physiol Lung Cell Mol Physiol. 2009; 296:L648–56. 10.1152/ajplung.90270.2008 19201816

[56] Schmittgen TD, Livak KJ. Analyzing real-time PCR data by the comparative C(T) method. Nat Protoc. 2008; 3:1101–8. 10.1038/nprot.2008.73 18546601

